# Machine-Learning Exploration of Exposure-Effect Relationships of Cisplatin in Head and Neck Cancer Patients

**DOI:** 10.3390/pharmaceutics14112509

**Published:** 2022-11-18

**Authors:** Céleste Cauvin, Laurent Bourguignon, Laure Carriat, Abel Mence, Pauline Ghipponi, Sébastien Salas, Joseph Ciccolini

**Affiliations:** 1 Clinical Pharmacokinetics, La Timone University Hospital of Marseille, 13005 Marseille, France; 2 COMPO Team, Centre de Recherche en Cancérologie de Marseille, Inserm U1068 Marseille, INRIA Sophia Antipolis, 06902 Valbonne, France; 3 Medical Oncology Unit, La Timone University Hospital of Marseille, 13005 Marseille, France

**Keywords:** cisplatin, machine-learning, Bayesian, pharmacokinetics, exposure-effect relationships

## Abstract

Background: Cisplatin is a pivotal drug in the treatment of head and neck cancer, and personalized dosage should help the preservation of an optimal toxicity–efficacy ratio. Methods: We analyzed the exposure-effect relationships of 80 adult patients with head and neck cancers and treated with standard Cisplatin-based regimen administered as three-hour infusion. Individual pharmacokinetics (PK) parameters of Cisplatin were identified using a Bayesian approach. Nephrotoxicity and ototoxicity were considered as typical Cisplatin-related toxicities according to Common Terminology Criteria for Adverse Events (CTCAE) standards. Efficacy was evaluated based upon Response Evaluation Criteria in Solid Tumors (RECIST) criteria. Up to nine different machine-learning algorithms were tested to decipher the exposure-effect relationships with Cisplatin. Results: The generalized linear model was the best algorithm with an accuracy of 0.71, a recall of 0.55 and a precision of 0.75. Among the various metrics for exposure (i.e., maximal concentration (Cmax), area-under-the-curve (AUC), trough levels), Cmax, comprising a range between 2.4 and 4.1 µg/mL, was the best one to be considered. When comparing a consequent, model-informed dosage with the standard dosage in 20 new patients, our strategy would have led to a reduced dosage in patients who would eventually prove to have severe toxicities while increasing dosage in patients with progressive disease. Conclusion: Determining a target Cmax could pave the way for PK-guided precision dosage with Cisplatin given as three-hour infusion.

## 1. Introduction

Cisplatin (cis-diamminedichloroplatinum (II) (CDDP)) is a mainstay to treat a variety of solid tumors [1], including head and neck cancer, in combination with cytotoxics, radiation therapy, or monoclonal antibodies. For several years Cisplatin has been administered as a 120-h continuous infusion to ensure optimal efficacy [2]. This long-lasting infusion has been successfully tested in head and neck cancer patients, especially when combined with the 5-FU that is usually given as well as a five-day continuous infusion in this setting [3]. In our institute, such long-lasting infusion enabled a real-time Bayesian adaptive dosage to be performed to reach a Cmax of 1.4 µg/mL, defined as the target exposure to be reached, to ensure an optimal toxicity/efficacy ratio [4,5]. Since the mid-2010’s, a new standard for Cisplatin has emerged with short-duration (i.e., three hours) infusion times every three weeks [6]. This is fully in line with a current trend for de-escalating treatments in head and neck cancer patients [7]. Such regimen allows patients to be treated in daily hospitalization units at the hospital, thus preserving quality of life, while being cost-effective. Of note, with such a three-hour infusion, it is not possible to personalize dosage in real-time anymore, as with the former five-day continuous infusion. However, determining individual PK parameters of Cisplatin during the first course could help in customizing the dosage of forthcoming administrations, provided that the pharmacokinetics (PK) parameters remain stable from one course to another, and that a target concentration to be reached in plasma has indeed been identified. The main side-effects due to cisplatin include kidney impairment, electrolyte disorders, myelotoxicity and ototoxicity [8,9].

In this work, we studied the exposure-effect relationships of Cisplatin administered as a three-hour infusion in head and neck cancer patients, as a means to determine the target metrics in exposure that could be used alongside adaptation of dosage in a prospective way.

## 2. Materials and Methods

### 2.1. Patients

Clinical and biological data from 80 real-world patients with head and neck cancer, hospitalized at the University Hospital of Marseille (France) between January 2021 and December 2022, were retrospectively collected. Patients (23 F, 57 M) were 58 years old (range: 15–85), with a mean body weight of 66 kg (range: 33–108) and a mean BSA of 1.76 m^2^ (range: 1.23–2.16). All patients were treated with 3 h intravenous infusion ranging from 75 to 100 mg/m^2^ Cisplatin (mean dose: 166.7 mg, range 63–200), repeated every 21 days for a minimum of three cycles, following standard guidelines in this setting [10]. All patients had stage-3 or stage-4 disease, since patients with stage 1–2 cancers are primarily treated with surgical resection. The various treatments associated with Cisplatin were radiation therapy for 60 patients, Cetuximab for 12 patients, 5-FU for 10 patients, Docetaxel for 2 patients, Nivolumab for 2 patients and Gemcitabine for 1 patient. For 4 patients, 5-FU was combined with Cetuximab, for 1 patient 5-FU was combined with Docetaxel and for 1 patient Cetuximab and Docetaxel were both combined. Treatment and sampling for biological analysis including drug monitoring, and clinical evaluations were all performed following standard practice in our institute in all patients with cancer. No extra-sampling and no modification of treatment, dosage or care was performed as part of this study.

### 2.2. Data Collection

Plasma concentrations of Cisplatin were monitored in our institute as part of standard care in patients with cancer. Cisplatin trough levels (i.e., immediately before infusion starts) and Cmax levels (i.e., immediately after the infusion stops) were measured using a fully validated inductively coupled plasma mass spectrometry (ICPMS) method. ICPMS allowed the monitoring of platinum metal over a 0.1–4 µg/mL calibration range, with a precision <15% and an accuracy of 15% following European Medical Agency standards [11]. The effects of sample dilution, long term frozen storage and quantitation in the presence of other drugs were also investigated and validated. Sample nebulization was performed using a concentric nebulizer for plasma. The major isotopes of platinum and iridium (internal standard) were monitored at m/z of 195 and 193, respectively. All analyses were performed on a 7850 ICP-MS Agilent (Agilent, Paris, France).

For each patient, data available from medical records were dosage history, Cisplatin plasma levels monitoring, baseline anthropometric data (e.g., sex, weight, height, body-surface area), creatinine levels at each cycle, plus a comprehensive list of co-medications such as Angiotensin-converting enzyme inhibitors, Angiotensin receptor blockers, non-steroidal anti-inflammatory drugs, smoking status and alcohol use, and the presence of hypoalbuminemia at baseline. Treatment-related clinical data were safety and efficacy.

Efficacy was assessed after 12 weeks of treatment. Standard RECIST 1.1. criteria were used to define the therapeutic response. Patients were then split into 2 subsets: clinical benefit was defined as patients showing either complete response, partial response, or stable disease, whereas patients with progressive disease or relapse were considered as non-responding. Tolerance was monitored after each course. Because the co-administered drugs or radiation therapy were likely to act as confounding factors, here, renal toxicity and ototoxicity were retained as the sole dose-limiting toxicities associated with Cisplatin administration. Consequently, nephrotoxicity was assessed according to the NCI CTCAE grading (i.e., increase of more than 50% in serum creatinine compared with baseline and/or increase in serum creatinine of more than 26 µmol/L compared with baseline). Because little ototoxicity was observed in our cohort, a composite variable combining therapeutic response and absence of nephrotoxicity was built next.

Data exploitation after anonymization was granted upon the non-opposition principle at the Assistance Publique Hôpitaux de Marseille (APHM) institute (i.e., unless the contrary is stated, all data collected during routine care can be used for biomedical research). Database query and subsequent data analysis were performed after approval by the Direction de la Recherche Clinique et de l’Innovation (DRCI) of the Assistance Publique Hôpitaux de Marseille under the MR-004 legal status [12].

### 2.3. Cisplatin PK Parameters Identification

Cisplatin PK was best described using a three-compartment model. Trough levels and Cmax levels of Cisplatin expressed as µg/mL plasma concentrations were used for Bayesian estimation of the individual PK parameters using a weighted least square estimator. Mean population parameters of Cisplatin were previously obtained using the standard 2-stages method [4]. No covariate was implemented in the final model. Reference population parameters were computed from 21 adult patients treated with Cisplatin for head and neck cancer and rich-data sampling (mean 1st, second and third macro-constants: A = 0.008106 L^−1^, B = 0.008095 L^−1^, and C = 0.006742 L^−1^; mean 1st, second and third rate constants: α = 0.5113 day^−1^, β = 0.07893 day^−1^, and χ = 0.05439 day^−1^). Using this population approach, it was possible to identify individual parameters of the forthcoming patients with only two sampling times (i.e., trough levels and Cmax) and the weighted least square algorithm in a Bayesian-estimation fashion. These included micro- and macro constants and eventually led to the estimation of standard PK parameters (Cl, Vd, Kel, T½). Quality of the estimates was evaluated by visual check for goodness-of-fit, the information quantity F and precision of the estimated parameters, which were all expected to be below 15% if they were to be accepted. All pharmacometrics analysis and Bayesian identification of individual PK parameters were performed using the KineticPro V1.0.3 software (IMMPS, Paris, France). 

### 2.4. Exposure Parameters

Individual PK parameters (i.e., clearance, Kel, Vd, T1/2) obtained after Bayesian identification were used to estimate the area under concentration curve (AUC) of Cisplatin. The mean Cmax, Cmin and AUC of the first three Cisplatin cycles were determined for each patient and were used as exposure parameters in the following analysis.

### 2.5. Machine Learning

The different exposure parameters (i.e., mean Cmin, Cmax, AUC) were compared to identify the parameters most strongly associated with the composite response criterion. To achieve this, different machine learning algorithms were used to model the response as a function of all available features, including exposure parameters, and were compared based on accuracy of the predictions. The best model build was then used to quantify the information provided by each variable with a permutation feature importance method. To assess which algorithm was the most appropriate to predict the clinical benefit, the AutoML node of KNIME (version 4.6.0) was used: this node can automatically train supervised machine learning models for binary classification. All available algorithms were used (except deep learning, which was not well suited for our data with small number of subjects): naive bayes, logistic regression, neural network (multi-layer perceptron with backpropagation), gradient boosted trees, decision tree, random forest, XGBoost trees, generalized linear model.

In a first step, a preprocessing of the data was done, with a one-hot encoding of the categorical variables, and a min-max normalization of numerical variables. The data were split into a train set (70%) and a test set (30%), with a stratified sampling technique on the response variable. In the AutoML mode, the following parameters were used: accuracy as metrics for auto selection, one-hot encoding of string columns, five folds in cross-validation, and 70% for the size of the training set partition. Default values were used for the other options.

The machine learning algorithms used in this study were supervised learning algorithms, suitable for problems where the available data consist of labeled examples. As no single learning algorithm works best on all supervised learning problems, several algorithms were considered to be tested in this step.

Logistic regression is a well-known classification algorithm, used for predicting binary dependent variables. The generalized linear model (GLM) is a generalization of ordinary linear regression, where the linear model is related to the dependent variable with a specific link function, and where the dependent variable can have arbitrary distribution.

Decision trees, and by extension random forests, are well suited to heterogeneous data (i.e., a mix between continuous and discrete variables), and can be used for both classification and regression tasks. The decision tree has the advantage of being simple to interpret and robust against co-linearity of features. The random forest is a collection of decision trees that are usually trained using a bagging method: the random forest gathers forecasts from many decision trees and predicts the target variable based on the majority of votes. For this reason, they are often more accurate than a single decision tree.

Neural networks are classifiers inspired by biological neural networks. They consist of a succession of elementary units (neurons) aggregated into layers. Each neuron receives as input the features of the database, or the outputs of the previous layer of neurons, and its output is the weighted sum of all the inputs. Widely used in image processing, they have the disadvantage of requiring large quantities of data for their learning.

Naive Bayes classifiers are probabilistic classifiers based on Bayes’ theorem and conditional probability, with an assumption of independence between the features. It is a supervised classifier well suited for solving multi-class prediction problems for categorical input variables, but less suited to continuous variables, which will need to be discretized before analysis.

Gradient boosted and XGBoost trees algorithms are meta-algorithms that combine weak predictors (typically, decision trees) in order to give a stronger (and more accurate) predictor model, but with the disadvantages of a lower intelligibility.

During the learning step, a tuning of different hyperparameters was made, through a five-fold cross validation, with a random search strategy. The optimized hyperparameters were: “Default probability” for the naive Bayes; “Step size” for the logistic regression; “Number of hidden layers” and “Number of hidden neurons per layer” for the neural network; “Min number records per node” for the decision tree, “Number of trees” for the gradient boosted trees; “Tree Depth”, “Number of models” and “Minimum child node size” for the random forest; “eta” and “max depth” for the XGBoost trees; “lambda” and “alpha” for the generalized linear model.

All trained models were then applied to the test sample, and the predictions of all models were scored against the ground truth and performance metrics were computed. The best model was selected based on accuracy metric.

In the final step, a measurement of the importance of each exposure variable in predicting the target variable was performed using a permutation method with the global feature importance node of KNIME. In this approach, the difference between the model performance score when using all the original features and the model performance score when using all the original features except one which was randomly permuted, is measured. The process is repeated for each feature. The same process was repeated five times, and the average decrease in accuracy was calculated for each feature, as well as the standard deviation. A sharp drop in accuracy indicates a strong importance of this feature in the prediction of the target variable.

### 2.6. Exploration of Exposure-Effect Relationships

Clinical benefit was defined as a positive clinical response after three cycles of Cisplatin, and the absence of severe nephrotoxicity or ototoxicity during the same period. 

In a first step, the different exposure parameters (i.e., mean Cmin, Cmax, AUC) were compared, to identify the parameters most strongly associated with the composite response criterion. To achieve this, nine different machine learning algorithms (i.e., naive Bayes, logistic regression, neural network, gradient boosted trees, decision tree, random forest, XGBoost trees, generalized linear model, and deep learning) were used to model the response as a function of all available features, including exposure parameters, and were compared based on accuracy of the predictions. The best model build was used to quantify the information provided by each variable with a permutation feature importance method. During this step, each variable was iteratively shuffled and the decrease in accuracy of the model’s predictions was determined. 

In a second step, the optimal therapeutic range was identified by determining the range of exposure that maximized the number of patients with positive outcomes when exposure was in the range and negative outcomes when exposure was outside of the range. Lower and upper bounds of the tested therapeutic ranges were generated with a Bayesian parameter optimization (i.e., tree-structured Parzen estimation, TPE). All statistical analyses were undertaken using R 4.2 [13] and KNIME [14].

### 2.7. Validation Set for Adaptive Dosage

**Figure 1:**
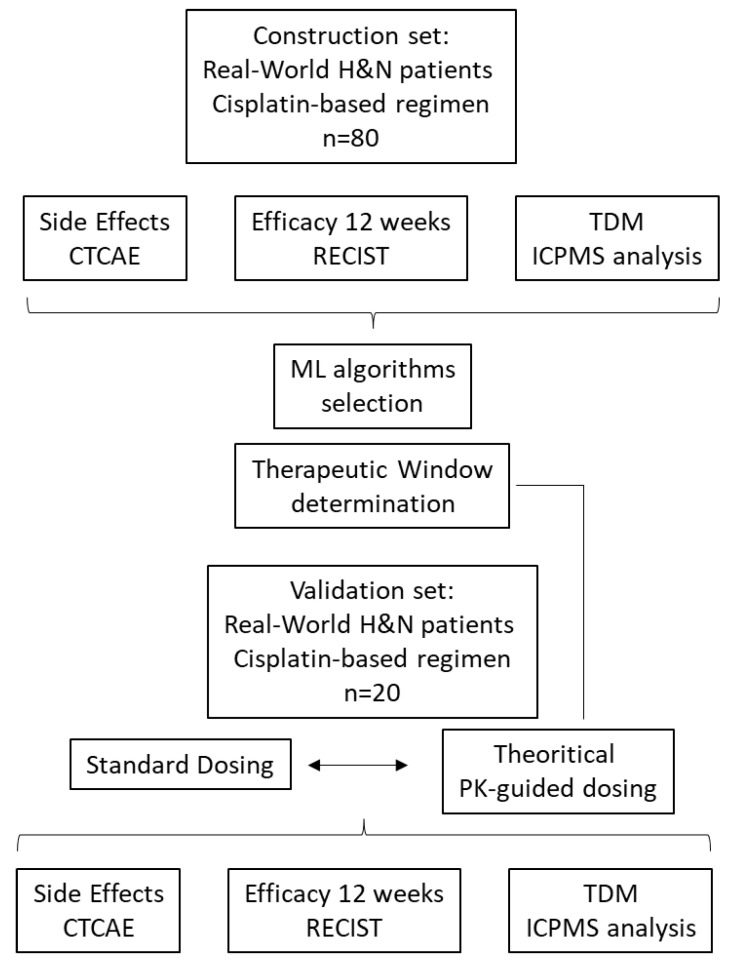
Workflow diagram. Data from 100 real-world patients were used (construction set: 80 patients, validation set: 20 patients).

Twenty new head and neck cancer routine patients scheduled for treatment with standard Cisplatin in our institute were used retrospectively as a validation set to evaluate the usefulness of the model. Basically, PK parameters were identified upon Course-1 as described previously. Once they were obtained, trough levels on D21 were simulated and a theoretical dose proposal was computed using the Kinetic Pro software for the forthcoming course, with respect to the therapeutic window previously identified. Differences between simulated trough levels and measured Cmin, between proposed dosage and actual dosage, and between desired exposure and final exposure after the second Course with respect to the therapeutic window upon standard dosage, were studied, as well as clinical outcomes (i.e., efficacy, toxicity). Figure 1 shows the workflow diagram of the proposed methodology.

## 3. Results

### 3.1. Exposure and PK Parameters

A total of 384 plasma concentrations were collected from the 80 patients. Pharmacokinetic and patients’ characteristics are summarized in Table 1.

**Figure 2:**
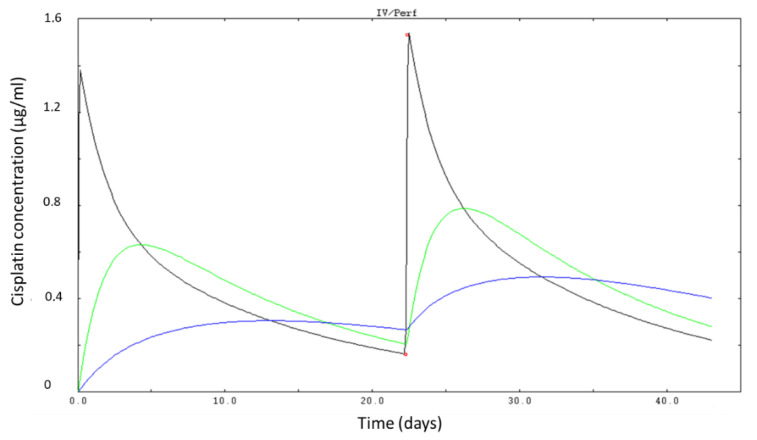
Time-concentration profile simulated after Bayesian identification of cisplatin pharmacokinetic parameters in a representative patient. Red dot: measured concentration in plasma. Black line: first compartment, green line: second compartment, blue line: third compartment (Kinetic Pro software caption).

The mean Cisplatin administered dose was 164 ± 27 mg (range 63–200, CV = 16%). The mean total clearance was 4.25 ± 1.33 L/h (range 0.18–10.8 L/h, CV = 31%), mean AUC was 41.96 ± 14.39 µg/mL/h (range 13–85 µg/mL/h, CV = 42%), mean trough level was 0.21 ± 0.17 µg/mL (range 0–0.65 µg/mL, CV = 81%) and mean Cmax was 3.26 ± 1.05 µg/mL (range: 1.12–46.7 µg/mL, CV = 32%). Figure 2 shows a time-concentration profile simulated with Kinetic Pro after Bayesian identification of individual pharmacokinetics parameters for a typical patient.

### 3.2. Clinical Data

In this work, nephrotoxicity and ototoxicity were the canonical Cisplatin-induced side-effects to be considered as safety metrics. Overall, 43 out of 80 patients (i.e., 54%) experienced severe toxicities (i.e., grade 3 and above, CTCAE grading). No treatment-related death was observed. Regarding specific Cisplatin-related toxicities, 30 patients (i.e., 37%) experienced such side-effects. Severe nephrotoxicity was observed in 17 patients (i.e., 21.3%) and 12 patients had severe ototoxicity (i.e., 15%). Regarding treatment efficacy, of the 80 patients studied, 65 patients (i.e., 81.2%) had clinical benefit whereas 15 patients (18.7%) had progressive disease.

### 3.3. Exposure-Effect Relationships

The machine-learning algorithm that best describe the relationship between available features and outcome was the generalized linear model, with an accuracy of 0.71, a recall of 0.45 and a precision of 0.83, as shown in Table 2. Using a permutation method on this model, Cmax was found to be the more useful exposure parameter to explain the response, with an accuracy reduction of 0.062 versus 0.05 and 0.013 for AUC and Cmin respectively.

### 3.4. Determination of the Cisplatin Therapeutic Range

The optimal range for the exposure variable (mean Cmax for the first three Cisplatin infusions), as determined with the TPE algorithm, was the interval the comprised the range between 2.1 µg/mL and 4.1 µg/mL. Patients whose Cmax values were within this range had a lower frequency of ototoxicity, a higher frequency of efficacy, but a slight increase in the frequency of nephrotoxicity, compared with patients whose Cmax values were outside this range. Overall, the proportion of patients with treatment success (efficacy without toxicity) was 64% for patients within the proposed therapeutic range, versus 42% for patients outside this range, as shown in Table 3.

### 3.5. Validation Set

**Figure 3:**
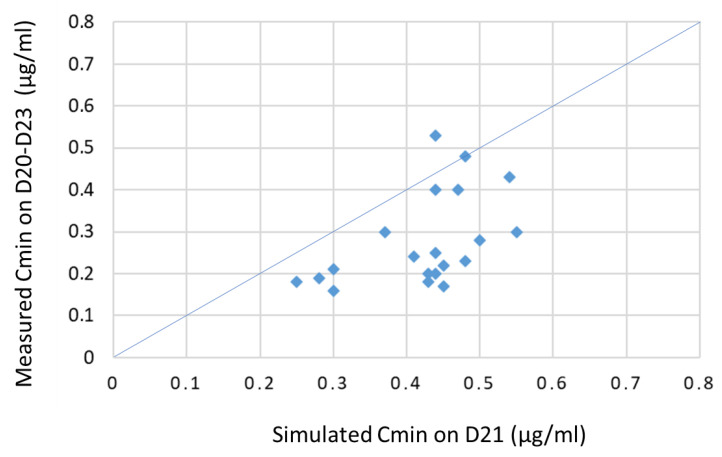
Actual vs. simulated trough levels in 20 patients. Measured Cmin were lower than simulated Cmin on D21 because the administration of most patients was performed up to 23 days after the previous course, thus lowering the measured concentration.

We compared the actual doses administered to 20 new head and neck cancer patients (8 F, 12 M, mean age: 60.4 ± 9.4 years) with the theoretical doses that would have been given by the model, based upon identification of PK parameters upon the first course and proposal for a personalized dosage to fulfil the requirements on the newly defined therapeutic window. Mean Cisplatin dose administered at the first course was 171.3 ± 28 mg (min: 119 mg, max: 200 mg). Mean Cmax measured after the first course was 2.9 ± 0.6 µg/mL (min: 1.46 µg/mL, max: 4.09 µg/mL). Only three out of 20 patients were strictly out of the therapeutic window (i.e., 1.46, 1.95 and 4.29 µg/mL). Mean Cisplatin clearance was 3.9 ± 0.3 L/H. Proposed dosage was calculated to reach the middle of the therapeutic window (i.e., 3.1 µg/mL) for the forthcoming courses. The mean proposed Cisplatin dose for the second course was 165.6 ± 30.2 mg (min: 93.8 mg, max: 224.5 mg). The actual administered Cisplatin doses for the second dose was close to the starting doses (i.e., 171 ± 27.8 mg). Trough levels measured immediately before the second administration starts were 0.3 ± 0.1 µg/mL. Of note, simulated trough levels on D21 were higher (i.e., 0.4 ± 0.1 µg/mL, *p* = 0.001, paired t-test) but this difference can be attributed to the fact that only three patients out of 20 were sampled for through levels on D21 sharp (range: 20.2–23 days) and that the actual D21 concentrations were thus not available for comparison since most patients were administrated later than D21. Figure 3 shows the correlation between the simulated Cmin and the measured Cmin in the validation set.

**Figure 4:**
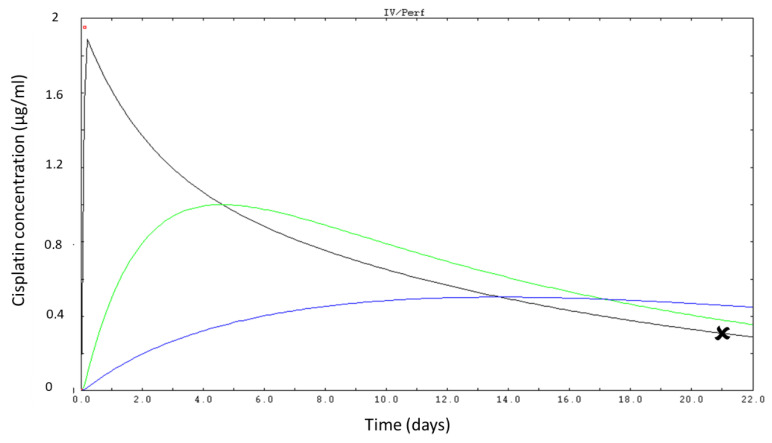
Simulation of plasma concentration of Cisplatin on D21 after Bayesian identification of cisplatin pharmacokinetic parameters in a representative patient. Red dot: measured concentration in plasma. Black line: first compartment, green line: second compartment, blue line: third compartment. Black cross: simulated plasma level on D21 (Kinetic Pro software caption).

Figure 4 displays a typical simulation of Cisplatin concentrations in the three compartments after the first course as performed on Kinetic Pro.

**Figure 5:**
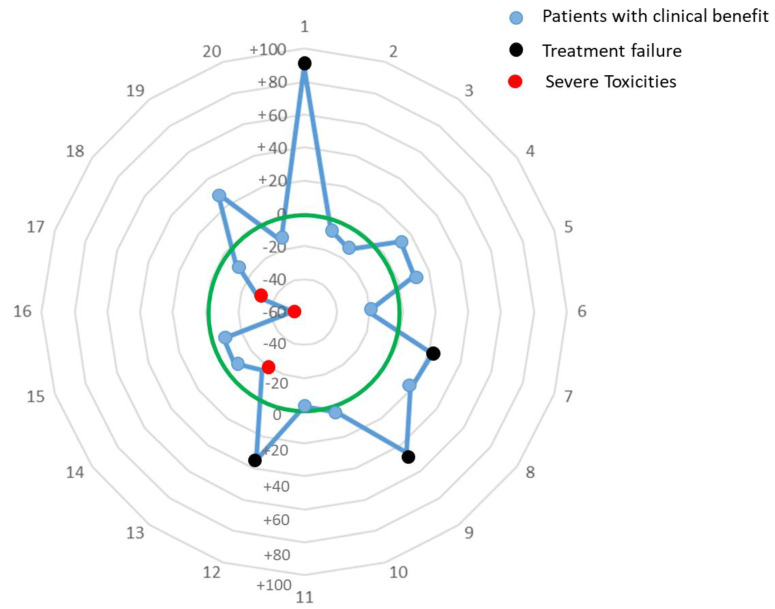
Model-based proposed changes (Y scale: % change as compared with initial dosage) in dosage after the first administration, and clinical outcome finally observed. Each dot represents a patient. Green Circle: No change in dosage. Outer green circle: our model proposed to increase dosage. Inner green circle: our model proposed to reduce the dosage. The four patients with progressive disease would have all been treated with higher doses, whereas the three patients with severe toxicities would all have been treated with a reduced dosage per model recommendations.

Out of the 20 patients, four patients had progressive disease (i.e., 20%), whereas three other patients (i.e., 15%) experienced severe Cisplatin-induced toxicities such as kidney failure. Mean Cmax recorded during the second course of Cisplatin administration was 2.4 ± 0.4 µg/mL for patients with progressive disease and 3.9 ± 0.5 µg/mL for the patients with severe toxicities. Of note, the three patients who experienced severe renal impairment upon treatment, were administered with Cisplatin doses of 183, 200 and 200 mg respectively. For those three patients, based upon individual PK parameters identified upon the first course, the Bayesian adaptive model would have proposed to instead administer 152.5 mg (i.e., −17% reduction), 139 mg (−30% reduction) and 115.8 mg (i.e., −53% reduction) respectively. Conversely, four other patients experienced progressive disease upon treatment, leading to three disease-related deaths for four of them. Those patients were treated with doses of 119, 124 and 173 and 179 mg of cisplatin, respectively. Based upon our Bayesian adaptive strategy, personalized dosage of 224.5 (i.e., +88% increase), 181 (i.e., +46% increase), 233 (i.e., +35% increase) and 218 mg (i.e., +22% increase) would have been proposed for the second course to reach the middle of the defined therapeutic window. For the 12 out of the 13 remaining patients with no severe toxicities and clinical benefit, our model would have proposed only slight changes in dosage ranging from −2.6 to +16.7% from the starting dose. For a last patient showing a Cmax after the first course of 2.15 µg/mL and who had complete response with no eventual severe toxicities, the model would have wrongly suggested to increase their dosage by 29% at the second administration. Figure 5 illustrates the % change in dosage for the 20 patients.

## 4. Discussion

Developing precision medicine is a major trend in oncology, especially with cytotoxics because of their narrow therapeutic index and interindividual variability. This calls for the implementation of tools for adaptive dosage, to ensure an optimal toxicity/efficacy ratio at bedside. Because most cytotoxics are administered via I.V., it is possible to precisely tune the dosage and to prepare a personalized infusion bag, as compared with oral anticancer drugs with limited options when customizing dosage. Cisplatin is given as a three-hour infusion in association with 5-FU and radiation therapy for treatment of head and neck cancer. Administration of 5-FU is partly personalized because of upfront DPD testing performed in all patients scheduled for fluoropyrimidine drugs in our institute [15]. Regarding Cisplatin, we previously developed an adaptive strategy when the drug was given as a 120-h infusion, thus enabling therapeutic drug monitoring, PK parameter identification and dose adjustment to be performed in real time throughout the infusion. Reduction in infusion-time of Cisplatin prevents such a sophisticated strategy from being carried out, however, it is still conceivable that Cisplatin PK parameters identified during a given course could be used alongside a customized dosage for the next course, provided that a target exposure has been identified as the therapeutic window, and that intra-patient variability is not too high from one course to another, i.e., a three-weeks period. The primary objective of this work was to describe the exposure-effect relationships in head and neck cancer patients treated with combinatorial therapies including three-hour infusion of Cisplatin.

The main safety concern with Cisplatin is kidney impairment, a typical dose-limiting toxicity [16]. In addition, ototoxicity is another concern frequently observed in Cisplatin-treated patients [17]. With respect to the fact that patients were treated with several anticancer agents, here we focused on those two typical Cisplatin-related side-effects, to avoid possible confounding factors due to the concomitant administration of 5-FU that is more likely to trigger digestive toxicities and myelotoxicities, Cetuximab that leads to skin-toxicity, or Docetaxel that is associated with neurotoxicity and neutropenia. To determine which exposure marker was most strongly associated with clinical response, different machine learning algorithms were compared prior to selecting the best model. Some algorithms tested were found to be poorly suitable for our data, leading subsequently to poor predictive performance. The clinical response was not perfectly predicted by all of the tested algorithms, as the available variables only partially explain this composite criterion. Nevertheless, this process made it possible to identify the exposure metrics as the most strongly explanatory of our target variables.

Of the various metrics for exposure tested (i.e., trough levels, Cmax, and AUC), Cmax was the most relevant, an observation that is in line with previous observations with 120-h continuous infusion [4]. Our statistical analyses suggest that Cmax levels comprising a range between 2.1 µg/mL and 4.1 µg/mL could be associated with better clinical outcome, i.e., patients less likely to develop Cisplatin-related toxicities while preserving the probability of achieving a clinical benefit.

In our cohort, the exposure parameter that seems to best explain treatment efficacy was Cmax, rather than through levels or AUC. Of note, the relevance of this variable is relative in comparison with other determinants of the response such as age or sex (reduction in accuracy of 0.15 and 0.125 respectively, versus 0.062 for Cmax). However, unlike sex or age, drug exposure is one of the few variables that can be controlled through dosage optimization. Still, it should be noted that the clinical benefit is only imperfectly described by the model used to assess the therapeutic range (accuracy of 0.71). Because of this, it is possible that the relative importance of the exposure parameters may be different in another cohort, or that a different optimal range of Cmax may be found.

When used retrospectively on routine patients, we found that this Bayesian adaptive dosage strategy could have maybe helped to improve clinical outcome. Out of the 20 patients we monitored, the three patients who experienced severe renal toxicities would have been treated with reduced Cisplatin dosage based upon the first course identification of PK parameters, whereas conversely the four patients with progressive disease would indeed all have been treated with higher Cisplatin dosage. It is not possible to draw any conclusion from this observation, because we do not know whether such changes in dosage would have been sufficient to translate next into a different clinical outcome in those patients. However, it does suggest that the toxicity/efficacy ratio could maybe have been improved for those patients by a shift towards a personalized dosage as soon as the second course. Conversely, our model would have wrongly proposed to increase the dosage for a patient who showed no toxicities and an eventual clinical benefit, therefore possibly triggering toxicities and thus highlighting the limits of our approach. In addition, this kind of Bayesian adaptive dosage strategy postulates that individual PK parameters remain stable from one course to another—which may not necessarily be true for every patient with respect to the heavy polypharmacy, the evolving comorbidities in the elderly, and the impact cisplatin has on renal function.

Overall, our proposed range of Cmax values could be considered as a possible target therapeutic window for Cisplatin in combination with chemotherapy in head and neck cancer patients treated with three-hour infusion. It must be kept in mind that although it is the best of the exposure metrics we tested, Cmax is by far less relevant than sex or age, but unlike these, Cmax is an actionable parameter through adaptive dosage strategies. Of note, numerous population PK parameters with cisplatin have been published by others [18] including in specific populations [19]. In this respect, the adaptive Bayesian dosage strategy we present here can be easily implemented at bedside, provided that TDM is performed and that basic pharmacometrics can be undertaken. Because Bayesian estimation allows the identification of individual parameters, it will be possible to simulate, after the first course, the trough levels reached by the patient 21 days later, i.e., just before starting the new administration of Cisplatin. Assuming that individual clearance values will not markedly change over this period, and with the 2.1–4.1 µg/mL range as a target Cmax to be reached, it will be possible to calculate a personalized dosage for the next courses as a means to limit the risk of Cisplatin-induced side effects and to improve efficacy.

## 5. Conclusions

Implementing precision medicine in oncology is a major trend. Developing adaptive dosage strategies with cytotoxics should help ensure an optimal toxicity/efficacy ratio. Despite being an old drug, Cisplatin is the pivotal drug for a variety of regimen. Here we have tentatively established a therapeutic window for this drug (i.e., Cmax), to be reached to limit the incidence of Cisplatin-related toxicities while preserving clinical efficacy. Bayesian-based adaptive dosage should help a following customized Cisplatin dosage from one course to another, based upon only two PK samples (i.e., Cmin and Cmax), thus enabling precision dosage with this canonical drug.

## Figures and Tables

**Table 1:** Patients’ characteristics. SD: standard deviation. All patients were hospitalized for head and neck cancer.

Characteristics	Mean ± SD
Number of patients	80
Gender (Female/Male)	23/57
Age (years)	58 ± 11
Body weight (kg)	65.5 ± 13
BSA (m^2^)	1.76 ± 0.19
Dose (mg)	164 ± 27
Cl (L/h)	4.2 ± 1.3
AUC (µg/mL/h)	42.0 ± 14.4
C_o_ (µg/mL)	0.21 ± 0.17
Cmax (µg/mL)	3.3 ± 1.0
C_predicted_ (µg/mL)	0.41 ± 0.15
Creatinine (µMol/L)	72.3 ± 18.7

**Table 2:** Scoring metrics of machine learning algorithms.

Model	Accuracy	Recall	Precision
Generalized Linear Model	0.71	0.55	0.75
Naive Bayes	0.67	0.27	1
Random Forest	0.67	0.27	1
Neural Network	0.63	0.55	0.63
Decision Tree	0.63	0.55	0.6
Gradient Boosted Trees	0.58	0.36	0.57
Logistic Regression	0.58	0.36	0.57
XGBoost Trees	0.5	0.18	0.4

**Table 3:** Number and proportion of patients with toxicity, therapeutic response, or therapeutic success (efficacy without toxicity) according to Cmax values.

		Within Therapeutic Range (*n* = 44)	Outside Therapeutic Range (*n* = 36)
Nephrotoxicity	yes	11 (25%)	6 (17%)
	no	33 (75%)	30 (83%)
Ototoxicity	yes	4 (9%)	8 (22%)
	no	40 (91%)	28 (78%)
Efficacy	yes	39 (89%)	26 (72%)
	no	5 (11%)	10 (28%)
Clinical Benefit	yes	28 (64%)	15 (42%)
	no	16 (36%)	21 (58%)

## Data Availability

Data will be made available upon request to the corresponding author.
